# STRIDE-DB: a comprehensive database for exploration of instability and phenotypic relevance of short tandem repeats in the human genome

**DOI:** 10.1093/database/baae020

**Published:** 2024-04-11

**Authors:** Bharathram Uppili, Mohammed Faruq

**Affiliations:** Genomics and Molecular Medicine, CSIR-Institute of Genomics and Integrative Biology (CSIR-IGIB), Delhi 110007, India; CSIR-HRDC Campus, Academy for Scientific and Innovative Research, Ghaziabad 201002, India; Genomics and Molecular Medicine, CSIR-Institute of Genomics and Integrative Biology (CSIR-IGIB), Delhi 110007, India

## Abstract

Short Tandem Repeats (STRs) are genetic markers made up of repeating DNA sequences. The variations of the STRs are widely studied in forensic analysis, population studies and genetic testing for a variety of neuromuscular disorders. Understanding polymorphic STR variation and its cause is crucial for deciphering genetic information and finding links to various disorders. In this paper, we present STRIDE-DB, a novel and unique platform to explore STR Instability and its Phenotypic Relevance, and a comprehensive database of STRs in the human genome. We utilized RepeatMasker to identify all the STRs in the human genome (hg19) and combined it with frequency data from the 1000 Genomes Project. STRIDE-DB, a user-friendly resource, plays a pivotal role in investigating the relationship between STR variation, instability and phenotype. By harnessing data from genome-wide association studies (GWAS), ClinVar database, Alu loci, Haploblocks in genome and Conservation of the STRs, it serves as an important tool for researchers exploring the variability of STRs in the human genome and its direct impact on phenotypes. STRIDE-DB has its broad applicability and significance in various research domains like forensic sciences and other repeat expansion disorders.

**Database URL**: https://stridedb.igib.res.in.

## Introduction

In the field of genetics, our understanding of DNA has revealed the presence of repetitive elements, which can be broadly categorized into two distinct groups: tandem repeats and interspersed repeats. Interspersed repeats, often referred to as transposable elements (TEs), utilize flanking sequences to migrate within the genome, while tandem nucleotide repeats (TNRs) are genomic regions where repeating units are positioned adjacent to one another. These tandem repeats are further classified based on the size of the repeating unit, with satellites (>60 base pairs), minisatellites (10–60 base pairs) and microsatellites (<9 base pairs) being the primary divisions (1). Among TNRs, microsatellites/Short Tandem repeats (STRs) are simple sequence repeats of DNA consisting of 1–6 nucleotides that are widely distributed throughout the human genome. It is estimated that there are approximately 1.5 million STRs in the human genome, accounting for around 3% of the genome’s sequence (2, 3).

In the human genome, repetitive elements, such as microsatellites and STRs, have played prominent roles in association studies, resulting in the widely replicated discoveries of genes related to conditions like type 2 diabetes (TCF7L2) and prostate cancer (the 8q21 region) (4). STRs are highly polymorphic regions and can be used as genetic markers for a variety of applications, including disease genomics, population genetics and yet unidentified novel association with human pathologies and physiologies (5). The length of STRs can vary between individuals and populations, which makes them useful for identifying genetic diversity and relationships (6). However, the sequencing of STR regions presents a significant challenge due to the repetitive nature of these sequences.

The STRs were first connected to human diseases like spinobulbar muscular atrophy and fragile X syndrome in 1991 (7, 8). This discovery created the opportunity to investigate the relationship between repeat expansion in the genome and undiagnosed disorders.

In the past three decades, after the era of Next-Generation Sequencing (NGS), deciphering the repetitive region in genome has gone high, resulting in a new era of repeat expansion research revealing more than 50 STRs in genome to date, reporting for various Central Nervous System (CNS) disorders (9). Short-read sequencing technologies, such as Illumina and Ion Torrent, have revolutionized the way repeat sequencing is performed. However, their inherent limitations in read length and GC-bias have made accurate repeat analysis challenging. As a result, long-read sequencing technologies such as PacBio and Nanopore have emerged as viable alternatives for comprehensive repeat analysis. Since these technologies have become more affordable, they were now used as a diagnostic technique in various disorders like Friedreich’s Ataxia and Spinocerebellar Ataxias (10, 11). The accuracy on finding the number of repetitive units has improved the clinical diagnosis. However, using NGS-based whole exome analysis gives the yield of 30–40% covering conventional mutations (SNVs and small InDels) for Mendelian disorder, while the unexplained cases prompt the exploration of other genetic mechanism like tandem repeat instability for the missing heritability (12).

In a study published in 2021, Mitusushai *et al*. found that even in the general population, known pathogenic repeats in the genome were highly polymorphic (13). Consequently, it has become necessary to examine the repeats to further question the genomic features that cause the instability of the repeat elements. Literatures have been published to explore the possibility of Alu element near GAA repeat region of FXN gene for the instability of the repeat. So, it is important to know the Alu elements near the TNRs because mutations that occur during the reverse transcription of the Alu element can initiate and expand a Tandem Repeat, effectively linking the Alu element and TNR (14).

This valuable resource offers insights into the functional implications of STR variability and enhances our understanding of the genetic factors contributing to the phenotypic variation. The conservation of the sequence across 44 vertebrates gives more insight on the evolutionary possibility of instability of the repeat. The information from STRIDE-DB can be used to build novel diagnostic tools and targeted therapeutics, as well as to gain a better understanding of the significance of STRs for human pathologies and physiology.

## Materials and methods

### STRs in genome

We used hg19 version of the reference genome from the UCSC (http://hgdownload.cse.ucsc.edu/goldenpath/hg19/bigZips/). To find the STRs in the genome, the RepeatMasker tool was used (15). We used –noint option to get the tandem repeats present under the interspersed repeats and -no_is option was used to remove masking of bacterial insertion elements. And we tried with different -div values ranging from 10% to 50% and fixed with 50% deviation which yielded 1 551 701 repeats from the genome. The repeats were then annotated using Gene Transfer format (GTF) file from the gencode to know the functional location.

### Data resources of STRIDE-DB

#### 1000 genome STR data

The STR-genotyped VCF was taken from the 1000 genome project’s phase 3 (ftp://ftp.1000genomes.ebi.ac.uk/vol1/ftp/phase3/) (16). All the 2504 samples’ metadata were collected from the 1000 genomes project’s sample info file: 20130606_sample_info.txt. The VCF file was further processed using bcftools (v 1.9) by utilizing the super population information from the metadata, and the count for each STR variability in each repeats was calculated (17).

#### GWAS and ClinVar data

The genome-wide association studies (GWAS) data were downloaded from GWAS catalogue (data downloaded by September 2022), and the ClinVar data were downloaded from ClinVar ftp link (https://ftp.ncbi.nlm.nih.gov/pub/clinvar/tab_delimited/variant_summary.txt.gz) (September 2022).

#### Alu elements

We used RepeatMasker to identify the Alu elements across the genome and the data were intersected across the STRs.

#### Conservation score

We utilized phastCons score of 46 vertebrates from the UCSC genome browser and overlapped with the STRs using bedtools to get the conservation scores across the STR region.

#### Haploblock calculation

The Haploblock for each population of 1000 genome was calculated using plink (parameters of lower confidence interval of 0.7 and max-kb of 2000 were used).

**Figure 1.**
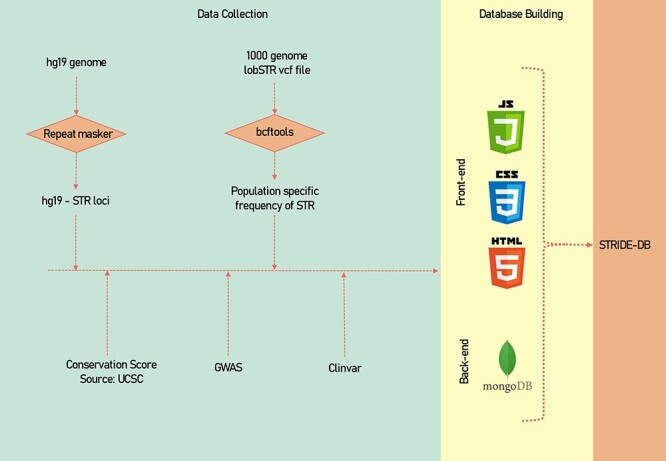
A systematic methodology of the data collection and data processing of STRIDE-DB.

A descriptive image of the methodology of data collection and data processing has been shown (Figure 1).

### Database implementation

The database has been built using PHP5, and Apache2 was used for local hosting and development. JavaScript was used for more fluid experience in filtering and showcasing the data. Plotly graphs were used to display necessary figures like different repeat configuration in each gene and to show the repeat variability among the population (18). The MongoDB (v 5.0.3) was used as a database for storing the data.

**Figure 2.**
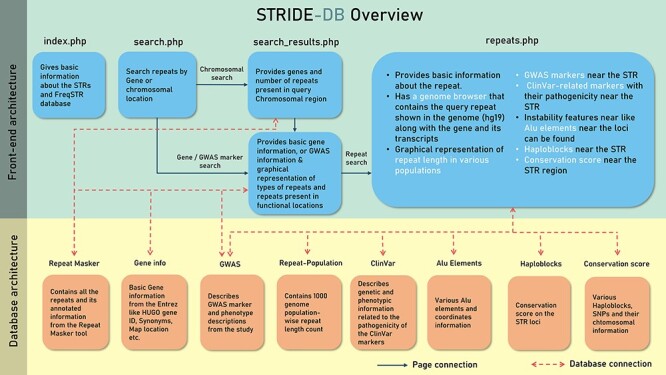
A systematic outline of the front-end and back-end architecture of STRIDE-DB.

The data were stored in five collections or tables under STRIDE-DB database (Figure 2). The pages were made based on the accessibility of the user query, which is either chromosomal location or genes to search for the repeats. Then repeats.php was made to show the repeat variability among the population and to understand the relevant phenotypes using GWAS and ClinVar.

## Results

### Database content and usage

#### Basic information

This web page is the gateway to the STRIDE-DB database. Users are given a brief description of STR as well as basic database information. This details the purpose of the database and displays general statistics of the various STRs in the genome(s).

#### Search and results page

The search term on this page is flexible and can take various semantic inputs, e.g. official gene name, chromosomal locations or GWAS loci.

**Figure 3.**
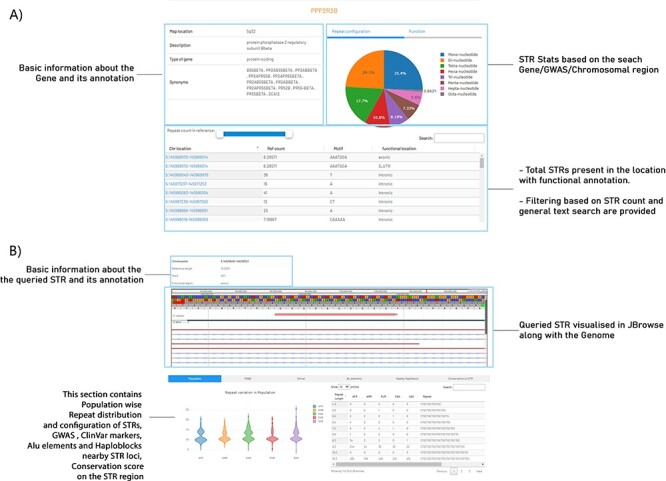
An example view of a query gene and an STR search in STRIDE-DB. (**A**) The resultant page of gene-wise search in STRIDE-DB for *PPP2R2B* gene. (**B**) Describing the selected STR from the *PPP2R2B* gene with genome browser visualization, population-wise repeat distribution, GWAS, ClinVar, Alu elements and Haploblocks nearby STR loci and conservation score on the STR region.

In the search page, a basic overview of the STRIDE-DB was provided. Further upon the search, this webpage provides users with gene information and the number of STR embedded in the respective genomic region. Further, the page provides information about the basic gene information, various STRs located in it, their locations, i.e. intronic, exonic and their respective frequencies (Figure 3A). The webpage also provides a slider to restrict the search results about a particular repeat count or a range of its counts. The display results can also be customized based on the type of repeats (di, tri, tetra, etc.).

#### Repeat information

User can select any region of interests and/or repeats and then it will take it to the information about that repetitive region in detail. The resulting search will provide information about basic repeat count (in the reference genome hg19), motif and the function region location (Figure 3B). Next section of the webpage is JBrowse plugin (19). This feature enables the user to browse the repeat in the hg19 genome. Three tracks which were preloaded into the plugin were the reference genome (hg19), the repeats from the RepeatMasker bed file and then the gene information from the GTF file from Ensembl (GRCh37). An option has been provided to browse in full screen mode, where users can input their BAM/VCF/BED files into the tracks and perform the analysis.

The next section of the website is divided based on six important features.

##### The population frequency of the repeats:

This describes the repeat count diversity among various 1000 genome populations. The violin plot describes the variability of the number of repeats in different population. This plot also shows different statistics, like max, min, median, mean, different quantile values of the repeats present in each population. A table is provided to analyse the different repeat configuration present in different world population, such as African, American, South Asian, East Asian and European.

##### GWAS:

Another important feature of this page was to understand and compare the phenotype caused by the variability of the repeats, by using the flanking GWAS markers. With default and maximum cutoff of 50 kb, the GWAS markers along with the disease trait, *P*-value from the study, SNP ID, and whether the repeat is present in upstream/downstream to the repeat region, are all fetched from the database collection and listed in the table. An option to search GWAS markers or to query phenotypes is provided. The data can be filtered using varied flanking regions like 5 kb, 10 kb and 50 kb. The GWAS study is also mentioned for reference, and a link is provided to redirect to the PubMed article.

##### ClinVar:

This feature provides various markers present in 50-kb flanking region (default cutoff) of the STR loci and describes the various pathogenic classifications from the ClinVar, and this facet helps to identify any other pathogenic phenotype that can be associated to repeat variability of the queried repeat. And the data filtering option was set like the previous, from 5 kb to 50 kb.

##### Alu elements:

Further, to explore the instability of the repeats, Alu elements were shown with a flanking cutoff of 2 kb. With this, users can explore the possibilities of Alu elements, and they can investigate the relation to the instability of the repeats.

##### Haploblocks:

This offers a list of Haploblocks across different 1000 genome population found within 50 kb flanking to the STR. This provides a valuable resource for haplotyping a STR region to find the repeat’s genetic structure, population history and potential functional implications of the STR region.

The tables in ClinVar, GWAS, Alu elements and Haploblocks are downloadable in Excel, CSV, PDF format, and an option to print table is also provided, making it user friendly.

##### Conservation score:

This provides a graphical representation of the conservation score calculated in the STR region. This helps us to understand the evolutionary stability of repeat.

## Discussion

### Known expansion-prone STR loci

A total of 54 known STR loci have been identified for various neuromuscular disorders, and all those has been catalogued under search.php for easy access to know the population repeat count frequency and the nearby GWAS and ClinVar loci.

### Redefining phenotypic variability by genetic correlates using STRIDE-DB for Down syndrome

Genetic markers for Down syndrome in which the heterozygosity has been explored in the North Indian population were D21S1435 (21:27 848 896–27 848 976—CTAT repeat), and D21S1411 (21:44 160 670–44 160 967—GATG repeat), with heterozygosity of 70.1% and 93.6%, respectively (20). When we analysed these regions in the STRIDE-DB, within 50-kb of these markers, there were GWAS studies which were associated to cholesterol level, heart rate response to beta blockers and asthma-related disorders found, which also coincides with the symptoms/effects of Down syndrome (Supplementary Table S1) (21–24), thus suggesting that these variations in the STRs can act as a clinical marker in screening Down syndrome before pregnancy.

Similarly, a study reporting D18S51 (18:60 948 900–60 949 006—AGAA repeat) having an association hypothesizes that the occurrence of a chronic disease depends on a programmed onset pattern (25). They showed a divergence in the incidence of lung cancer between carriers of the D18S51-20 allele and non-carriers. So when we analysed the region, within a 50-kb distance, a study reporting rs139419907 marker as a causative marker for lung cancer was found (26).

### Understanding forensic markers using STRIDE-DB

Eight forensic markers, which were associated to various traits such as male impulsive violent behaviour, crime of rape, hypertension, initiative aggressive behaviour and gastric cancer, were taken to understand the frequency in the control population. We found the risk allele for all markers found to be very rare in the population (Supplementary Table S2).

Therefore, in conclusion, this database provides easy access to information on STR loci throughout genome and their frequency in different population along with the possible phenotypes, allowing a better understanding of the genetic correlates of various disorders. Since this database was built using short-read sequencing data, longer STRs are still inaccessible. Therefore, through long-read sequencing technologies such as nanopore, we can estimate repeat length for longer STRs in different ethnicity, which may aid in better understanding their potential roles in disease and genetic diversity.

### Comparison of STRIDE-DB with other STR databases

Various databases like STRbase, WebSTR and CAGm have been used as a valuable resource for knowing STR information. STRBase mainly focus on STR markers which are reported and mainly built for forensic genomic science and STR typing kits (27). WebSTR, on the other hand, covers the genome-wide STRs and population information from 1000 genome population (28).

CAGm database created by Kinney *et al*. is a rich resource that catalogues polymorphic 625k microsatellites as observed across the 1000 genome population and covers the basic understanding of the variability of length of STRs and other features for data extraction and analysis with respect to the genotype information (29).

In contrast, STRIDE-DB provides a spectrum of features to understand these variations in the general population from a phenotypic perspective. STRIDE-DB fills in the gaps by allowing the researchers to determine the location and frequency of STRs quickly and efficiently in the human genome, as well as to study the potential link between these polymorphisms and various traits such as illness susceptibility and demographic ancestry. It also provides information of instability-causing features like Alu elements and Haploblocks, enabling researchers to assess the integrity and diversity of STRs in different population (Table 1).

**Table 1.** Comparison of STRIDE-DB features with other databases

Features	STRbase	WebSTR	CAGm	STRIDE-DB
STR-based features				
Abundance	Targeted forensic markers	Genome wide	Genome wide	Genome wide
No. of STRs	75	∼1700 K	∼625 K	∼1600 K
Dataset used	From literatures	KGP CRC-TCGA H3Africa	KGP	KGP
Annotation				
Gene	*N*	**Y**	**Y**	**Y**
Functional region annotation	*N*	**Y**	*N*	**Y**
Addition annotation				
GWAS	*N*	*N*	*N*	**Y**
ClinVar	*N*	*N*	*N*	**Y**
Conservation on STR loci	*N*	*N*	*N*	**Y**
Haploblock	*N*	*N*	*N*	**Y**
Expression correlation with gene	*N*	**Y**	*N*	*N*
Disease genomic STR loci	*N*	*N*	*N*	**Y**
Feature to download data	**Y**	**Y**	**Y**	**Y**
Genome browser	Y	*N*	*N*	**Y**
Database link	https://strbase-archive.nist.gov/index.htm	https://webstr.ucsd.edu/	http://www.cagmdb.org/	https://stridedb.igib.res.in/

^KGP: 1000 genome project; CRC: Colorectal cancer; Y: Yes; N: No.^

## Conclusion and future directions

Because of the sequencing limitations and complex nature of the STRs, it was difficult to study and explore these genomic elements. STRIDE-DB attempts to bridge the gap of STR variability-phenotype. This also provides a platform to understand the instability of these loci and understand its evolutionary significance. But variability of STRs among the population and STRs in the dark genome can be determined accurately by third-generation sequencing technologies, which gives more knowledge and ability to understand the STR’s benign and pathogenic facets in the genome.

## Supplementary Material

baae020_Supp

## Data Availability

The scripts used during Repeat Masker and the annotation of the STRs were mentioned in the link (https://raw.githubusercontent.com/bharathramh/STRIDE-DB_Codes/main/codes.md).
