# Supplementary material for: STRIDE-DB: a comprehensive database for exploration of instability and phenotypic relevance of short tandem repeats in the human genome
Source: Database (Oxford). 2024 Apr 11;2024:baae020. doi: 10.1093/database/baae020 (PMC11008502; doi:10.1093/database/baae020)
Supplement: baae020_Supp [file baae020_supp.zip › suppl_data/Supplementary table.docx]

**Supplementary table 1:** The GWAS markers from the STRIDE-DB database supporting to D21S1435 and D21S1411

| **Genetic marker** | **Position** | **Disease/Trait** | **P-value** | **SNPs** | **location from repeat** | **Study** |
| --- | --- | --- | --- | --- | --- | --- |
| D21S1435 | 21:27824285 | [Glucocorticoid receptor gene expression in B-cell precursor acute lymphoblastic leukaemia](http://www.ebi.ac.uk/efo/EFO_0600079), | 2.0E-6 | rs219649 | Downstream within 50 KB | [Inherited genetic variants associated with glucocorticoid sensitivity in leukaemia cells.](https://www.ncbi.nlm.nih.gov/pubmed/33002292) |
| D21S1435 | 21:27858939 | [HDL cholesterol levels](http://www.ebi.ac.uk/efo/EFO_0007805), | 9.0E-7 | rs71329836 | Upstream within 50 KB | [Gene discovery for high-density lipoprotein cholesterol level change over time in prospective family studies.](https://www.ncbi.nlm.nih.gov/pubmed/32109663) |
| D21S1435 | 21:27872161 | [Response to exercise (triglyceride level interaction)](http://www.ebi.ac.uk/efo/EFO_0007681), | 2.0E-6 | rs222158 | Upstream within 50 KB | [Genomic and transcriptomic predictors of triglyceride response to regular exercise.](https://www.ncbi.nlm.nih.gov/pubmed/26491034) |
| D21S1435 | 21:27873895 | [Heart rate response to beta blockers (atenolol monotherapy)](http://www.ebi.ac.uk/efo/EFO_0007766), | 3.0E-6 | rs2830261 | Upstream within 50 KB | [Genome-Wide Association Approach Identified Novel Genetic Predictors of Heart Rate Response to β-Blockers.](https://www.ncbi.nlm.nih.gov/pubmed/29478026) |
| D21S1411 | 21:44156769 | [Pulmonary function decline](http://www.ebi.ac.uk/efo/EFO_0004713), | 6.0E-6 | rs9979235 | Downstream within 50 KB | [Genome-wide association study of lung function decline in adults with and without asthma.](https://www.ncbi.nlm.nih.gov/pubmed/22424883) |

**Supplementary table 2 :** A use-case for Forensic applications using STRIDE-DB population frequency of 8 STR markers

| **STR marker** | **Genomic Region** | **STR unit count** | **AFR** | **AMR** | **EUR** | **EAS** | **SAS** | **Trait** | **Repeat count associated** | **P-Value** | **Reference** |
| --- | --- | --- | --- | --- | --- | --- | --- | --- | --- | --- | --- |
| TPOX | 2:1493389-1493620 | 7.8 | 0 | 0 | 0 | 0 | 0 | Crime of rape | 8, 12 | <0.05 | 1 |
|  |  | 11.8 | 0 | 2 | 0 | 0 | 0 |  |  |  |  |
| D5S818 | 5:123111185-123111333 | 6.5 | 1 | 0 | 0 | 1 | 0 | Gastric cancer | 13 combined with D8S1179-16 | <0.05 | 2 |
|  |  | 6.8 | 2 | 36 | 1 | 24 | 2 |  |  |  |  |
|  |  | 7.8 | 98 | 7 | 5 | 4 | 3 |  |  |  |  |
|  |  | 8.5 | 11 | 4 | 3 | 5 | 4 |  |  |  |  |
|  |  | 8.8 | 23 | 37 | 55 | 86 | 29 |  |  |  |  |
|  |  | 9 | 3 | 0 | 1 | 2 | 3 |  |  |  |  |
|  |  | 9.8 | 74 | 28 | 54 | 200 | 110 |  |  |  |  |
|  |  | 10 | 7 | 1 | 7 | 3 | 3 |  |  |  |  |
|  |  | 10.8 | 266 | 268 | 300 | 236 | 314 |  |  |  |  |
|  |  | 11.8 | 387 | 135 | 246 | 164 | 262 |  |  |  |  |
|  |  | 12 | 0 | 0 | 1 | 0 | 0 |  |  |  |  |
|  |  | 12.8 | 238 | 50 | 121 | 85 | 107 |  |  |  |  |
|  |  | 13.8 | 17 | 4 | 4 | 2 | 9 |  |  |  |  |
|  |  | 14.8 | 3 | 0 | 2 | 0 | 0 |  |  |  |  |
| CSF1PO | 5:149455735-149456053 | 4.3 | 2 | 0 | 0 | 0 | 0 | Male impulsive violent behaviour | 14 | 0.0035 | 3 |
|  |  | 5.3 | 3 | 0 | 0 | 1 | 0 |  |  |  |  |
|  |  | 6.3 | 6 | 1 | 1 | 6 | 1 |  |  |  |  |
|  |  | 6.8 | 91 | 2 | 3 | 6 | 2 |  |  |  |  |
|  |  | 7.3 | 13 | 10 | 14 | 16 | 8 |  |  |  |  |
|  |  | 7.5 | 2 | 0 | 1 | 0 | 0 |  |  |  |  |
|  |  | 7.8 | 105 | 9 | 2 | 1 | 2 |  |  |  |  |
|  |  | 8.3 | 12 | 14 | 16 | 22 | 10 |  |  |  |  |
|  |  | 8.8 | 70 | 14 | 28 | 50 | 20 |  |  |  |  |
|  |  | 9 | 0 | 0 | 0 | 0 | 1 |  |  |  |  |
|  |  | 9.3 | 13 | 11 | 11 | 22 | 20 |  |  |  |  |
|  |  | 9.8 | 280 | 148 | 265 | 215 | 191 |  |  |  |  |
|  |  | 10.5 | 0 | 0 | 0 | 0 | 1 |  |  |  |  |
|  |  | 10.8 | 297 | 171 | 257 | 216 | 259 |  |  |  |  |
|  |  | 11.3 | 1 | 0 | 0 | 0 | 0 |  |  |  |  |
|  |  | 11.8 | 252 | 211 | 254 | 278 | 308 |  |  |  |  |
|  |  | 12.8 | 58 | 35 | 56 | 69 | 43 |  |  |  |  |
|  |  | 13.8 | 9 | 0 | 4 | 8 | 14 |  |  |  |  |
|  |  | 14.8 | 0 | 0 | 2 | 4 | 0 |  |  |  |  |
| D7S820 | 7:83789392-83789617 | 5.8 | 1 | 1 | 0 | 0 | 3 | Intracerebral haemorrhage | 13 combined with D2S1338-18 | 0.023 | 4 |
|  |  | 6.8 | 10 | 13 | 16 | 1 | 36 |  |  |  |  |
|  |  | 7.5 | 2 | 0 | 0 | 0 | 0 |  |  |  |  |
|  |  | 7.8 | 220 | 49 | 154 | 116 | 176 |  |  |  |  |
|  |  | 8.5 | 1 | 0 | 0 | 0 | 2 |  |  |  |  |
|  |  | 8.8 | 112 | 55 | 86 | 43 | 37 |  |  |  |  |
|  |  | 9 | 0 | 0 | 0 | 3 | 0 |  |  |  |  |
|  |  | 9.5 | 0 | 0 | 2 | 0 | 0 |  |  |  |  |
|  |  | 9.8 | 287 | 132 | 178 | 80 | 171 |  |  |  |  |
|  |  | 10.5 | 1 | 1 | 1 | 1 | 0 |  |  |  |  |
|  |  | 10.8 | 160 | 123 | 124 | 270 | 139 |  |  |  |  |
|  |  | 11 | 0 | 0 | 3 | 0 | 0 |  |  |  |  |
|  |  | 11.5 | 0 | 0 | 0 | 2 | 0 |  |  |  |  |
|  |  | 11.8 | 77 | 99 | 77 | 121 | 103 |  |  |  |  |
|  |  | 12 | 0 | 0 | 1 | 0 | 0 |  |  |  |  |
|  |  | 12.8 | 11 | 19 | 25 | 11 | 13 |  |  |  |  |
|  |  | 13.8 | 2 | 6 | 3 | 2 | 0 |  |  |  |  |
| D8S1179 | 8:125907080-125907260 | 4.8 | 0 | 0 | 1 | 0 | 0 | Gastric cancer | 16 combined with D5S818-13 | <0.05 | 5 |
|  |  | 5.8 | 0 | 9 | 18 | 2 | 7 |  |  |  |  |
|  |  | 6.8 | 2 | 9 | 29 | 12 | 14 |  |  |  |  |
|  |  | 7.5 | 0 | 0 | 1 | 0 | 0 |  |  |  |  |
|  |  | 7.8 | 19 | 54 | 109 | 139 | 169 |  |  |  |  |
|  |  | 8.8 | 80 | 52 | 102 | 111 | 63 |  |  |  |  |
|  |  | 9.3 | 1 | 0 | 0 | 0 | 0 |  |  |  |  |
|  |  | 9.8 | 146 | 92 | 121 | 120 | 81 |  |  |  |  |
|  |  | 10.8 | 249 | 184 | 272 | 196 | 161 | Schizophrenia | 14 | 0.05 | 6 |
|  |  | 11.8 | 417 | 142 | 147 | 147 | 187 |  |  |  |  |
|  |  | 12 | 0 | 1 | 0 | 0 | 0 |  |  |  |  |
|  |  | 12.8 | 207 | 65 | 57 | 112 | 161 |  |  |  |  |
|  |  | 13.8 | 56 | 8 | 13 | 40 | 61 |  |  |  |  |
|  |  | 14.8 | 9 | 0 | 5 | 5 | 4 |  |  |  |  |
|  |  | 15.8 | 2 | 0 | 1 | 4 | 0 |  |  |  |  |
| TH01 | 11:2192220-2192381 | 3.8 | 1 | 1 | 0 | 0 | 0 | Male impulsive violent behaviour | 10 | <0.0001 | 7 |
|  |  | 4.8 | 14 | 9 | 8 | 4 | 5 |  |  |  |  |
|  |  | 5.5 | 0 | 0 | 3 | 0 | 0 |  |  |  |  |
|  |  | 5.8 | 194 | 186 | 208 | 102 | 255 |  |  |  |  |
|  |  | 6.8 | 489 | 196 | 175 | 285 | 146 |  |  |  |  |
|  |  | 7.8 | 271 | 50 | 94 | 58 | 120 |  |  |  |  |
|  |  | 8.5 | 1 | 0 | 7 | 1 | 0 |  |  |  |  |
|  |  | 8.8 | 160 | 70 | 156 | 333 | 242 | Hypertension | 9.3, 10 | <0.0005 | 8 |
|  |  | 9 | 0 | 0 | 0 | 1 | 1 |  |  |  |  |
|  |  | 9.3 | 0 | 0 | 0 | 0 | 1 |  |  |  |  |
|  |  | 9.5 | 79 | 115 | 245 | 46 | 116 |  |  |  |  |
|  |  | 9.8 | 5 | 3 | 6 | 25 | 8 |  |  |  |  |
|  |  | 10.8 | 0 | 0 | 0 | 1 | 0 |  |  |  |  |
| D13S317 | 13:82722059-82722243 | 6.8 | 0 | 1 | 1 | 2 | 10 | Intracerebral haemorrhage | 11 combined with vWA-17 | 0.021 | 9 |
|  |  | 7.8 | 23 | 61 | 127 | 305 | 195 |  |  |  |  |
|  |  | 8.8 | 12 | 107 | 89 | 108 | 97 |  |  |  |  |
|  |  | 9.8 | 321 | 175 | 291 | 262 | 254 |  |  |  |  |
|  |  | 10.8 | 0 | 0 | 0 | 0 | 0 |  |  |  |  |
|  |  | 11.8 | 533 | 146 | 202 | 74 | 185 |  |  |  |  |
|  |  | 12.8 | 150 | 52 | 54 | 19 | 52 |  |  |  |  |
|  |  | 13.8 | 45 | 26 | 16 | 4 | 27 |  |  |  |  |
|  |  | 14.8 | 2 | 0 | 0 | 0 | 2 |  |  |  |  |
| D19S433 | 19:30417027-30417232 | 7.8 | 2 | 0 | 0 | 3 | 1 | Initiative aggressive behaviour | 14.2 | 0.0011 | 10 |
|  |  | 8.8 | 16 | 0 | 0 | 0 | 0 |  |  |  |  |
|  |  | 9.8 | 87 | 4 | 5 | 5 | 0 |  |  |  |  |
|  |  | 10.8 | 155 | 41 | 70 | 47 | 66 |  |  |  |  |
|  |  | 11.8 | 519 | 266 | 386 | 388 | 392 |  |  |  |  |
|  |  | 12.8 | 0 | 0 | 0 | 0 | 0 |  |  |  |  |
|  |  | 13.8 | 57 | 53 | 78 | 67 | 49 |  |  |  |  |
|  |  | 14.8 | 5 | 6 | 7 | 8 | 26 |  |  |  |  |
|  |  | 15.8 | 1 | 0 | 0 | 0 | 0 |  |  |  |  |
|  |  | 16.8 | 0 | 0 | 0 | 0 | 0 |  |  |  |  |

*Red color signifies the risk allele in the population according to the reference

References:

1. Yang, C., Huajie, B., Gao, Z., Lin, Z., Zhao, H., Liu, B., et al. (2010). Association study between the genetic polymorphism of 15 STR loci and the crime of rape. Chin. J. Behavioral Medicine and Brain Science 19(5), 421-424.
2. Hui, L., Liping, G., Jian, Y., and Laisui, Y. (2014). A new design without control population for identification of gastric cancer-related allele combinations based on interaction of genes. Gene 540(1), 32-36. doi: 10.1016/j.gene.2014.02.033.
3. Yang, C., Ba, H., Gao, Z., Zhao, H., Yu, H., and Guo, W. (2013a). Case-control study of allele frequencies of 15 short tandem repeat loci in males with impulsive violent behavior. Shanghai Arch. Psychiatry. 25(6), 354-363. doi: 10.3969/j.issn.1002-0829.2013.06.004.
4. Gai, L., Sun, C., Yu, W., and Liu, H. (2016). Screening of intracerebral hemorrhage associated allele combinations at different loci using a novel association analysis. Gene 579(1), 1-7. doi: 10.1016/j.gene.2015.12.031.
5. Hui, L., Liping, G., Jian, Y., and Laisui, Y. (2014). A new design without control population for identification of gastric cancer-related allele combinations based on interaction of genes. Gene 540(1), 32-36. doi: 10.1016/j.gene.2014.02.033.
6. Yang, G., Luo, H., Sun, W., and Liu, H. (2009). Relationship between polymorphism of gene D8S1179, D21S11, D18S51 and schizophrenia. J. Dalian Med. Univ. 31(5), 510-512.
7. Yang, C., Ba, H., Gao, Z., Zhao, H., Yu, H., and Guo, W. (2013a). Case-control study of allele frequencies of 15 short tandem repeat loci in males with impulsive violent behavior. Shanghai Arch. Psychiatry. 25(6), 354-363. doi: 10.3969/j.issn.1002-0829.2013.06.004.
8. Sharma, P., Hingorani, A., Jia, H., Ashby, M., Hopper, R., Clayton, D., et al. (1998). Positive association of tyrosine hydroxylase microsatellite marker to essential hypertension. Hypertension 32(4), 676-682. doi: 10.1161/01.HYP.32.4.676.
9. Gai, L., Sun, C., Yu, W., and Liu, H. (2016). Screening of intracerebral hemorrhage associated allele combinations at different loci using a novel association analysis. Gene 579(1), 1-7. doi: 10.1016/j.gene.2015.12.031.
10. Yang, C., Huajie, B., Haiying, Y., Gao, Z., Zhao, H., and Guo, W. (2013b). Association study of the genetic polymorphism of D2S1338 and D19S433 loci and the initiative-aggressive behavior in male population. Chinese J. Behav. Med. and Brain Sci. 1(2), 127-130.
